# Infant Trauma Management in the Emergency Department: An Emergency Medicine Simulation Exercise

**DOI:** 10.7759/cureus.316

**Published:** 2015-09-07

**Authors:** Sarah Mathieson, Desmond Whalen, Adam Dubrowski

**Affiliations:** 1 Discipline of Emergency Medicine, Memorial University of Newfoundland; 2 Emergency Medicine, Pediatrics, Memorial University of Newfoundland; 3 Marine Institute, Memorial University of Newfoundland

**Keywords:** emergency medicine, traumatic brain injury, infant trauma, pediatric emergency medicine, simulation-based medical education

## Abstract

In a trauma situation, it is essential that emergency room physicians are able to think clearly, make decisions quickly and manage patients in a way consistent with their injuries. In order for emergency medicine residents to adequately develop the skills to deal with trauma situations, it is imperative that they have the opportunity to experience such scenarios in a controlled environment with aptly timed feedback. In the case of infant trauma, sensitivities have to be taken that are specific to pediatric medicine. The following describes a simulation session in which trainees were tasked with managing an infantile patient who had experienced a major trauma as a result of a single vehicle accident. The described simulation session utilized human patient simulators and was tailored to junior (year 1 and 2) emergency medicine residents.

## Introduction

In trauma situations, it is imperative that situational awareness of patient status and projected course is maintained in an intense, dynamic environment. Cognitive load theory suggests that such intense and dynamic environments are suboptimal for learning novel and complex tasks [1-2]. However, evidence suggests that the use of simulation not only provides a safe, structured, and standardized environment for trainees to develop these skills without placing patients at harm, but also allows to reduce the complexity of the learning environment, subsequent cognitive load, and leads to better learning outcomes [3-6]. The effectiveness of simulation, when compared to other methods of instruction, has been noted among emergency medicine residents [7], with repeated simulation exposure resulting in even further improvement among students [8]. In situations, it is essential that teamwork, technical skills, and performance be all maximized. Simulation in pediatric medicine has been shown to increase team functioning, performance, and technical skills during trauma-based exercises [9]. The simulation also provides an opportunity to expose residents to pediatric trauma, a scenario in which they may have less clinical exposure during training.  

Following trauma, the steps taken to manage the patient and mitigate the severity of injuries sustained are essential to patient survival and outcomes. In infants, the management of trauma is different from that of adults owing in part to the differences in physiology and anatomy. For instance, in infants, the trachea is shorter and the larynx is anterior and cephalad, making airway management via intubation more difficult. Additionally, the mediastinum is more mobile and the chest wall more pliable, making tension pneumothorax and pulmonary contusions increasingly possible during intubation maneuvers as outlined by the Advanced Trauma Life Support (ATLS) protocol [10].

Pediatric emergency medicine poses its own challenges to the physician. Having an altered management plan to that of an adult, coupled to the emotional aspects of treating an infant, can result in a stressful environment for pediatric emergency room practitioners. The goal is to ensure that trainees will be adequately prepared to treat infant trauma, including the knowledge to manage the injuries in the appropriate way while at the same time acknowledging the emotional aspects of the situation, to guarantee affective thinking does not cloud medical management. Repeated exposure to this type of intense environment has been shown in the past to be effective in training emergency medicine residents to perform at a higher standard [8].

## Technical report

This simulation exercise was conducted in the Janeway Children’s Health and Rehabilitation Centre emergency room using the Gaudmard Noelle S575 human patient simulator (Gaumard Scientific, Miami, FL).

Prior to the simulation session, a detailed scenario template was provided to the simulation technical staff who programmed the mannequin and provided necessary materials and equipment for the exercise to be carried out effectively.

The scenario was designed to be used as a team learning activity with several confederates role-playing different healthcare professionals as required by the case. This scenario can be adapted to inter-professional learning if students from other disciplines are included and perform in their respective roles. The overall objectives of the simulation session, a general overview of the case, and the role of each individual participant were explained to the trainee(s) during the pre-scenario briefing.

**Figure 1:**
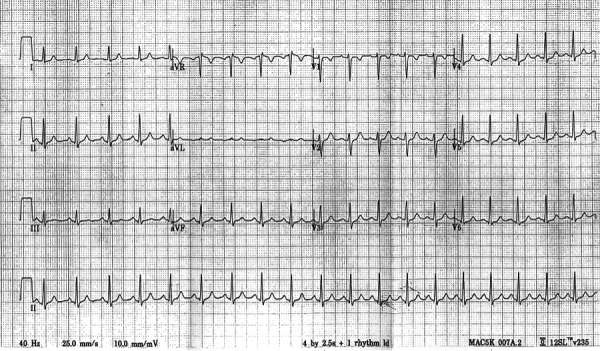
EKG to be provided to trainee on request

**Figure 2:**
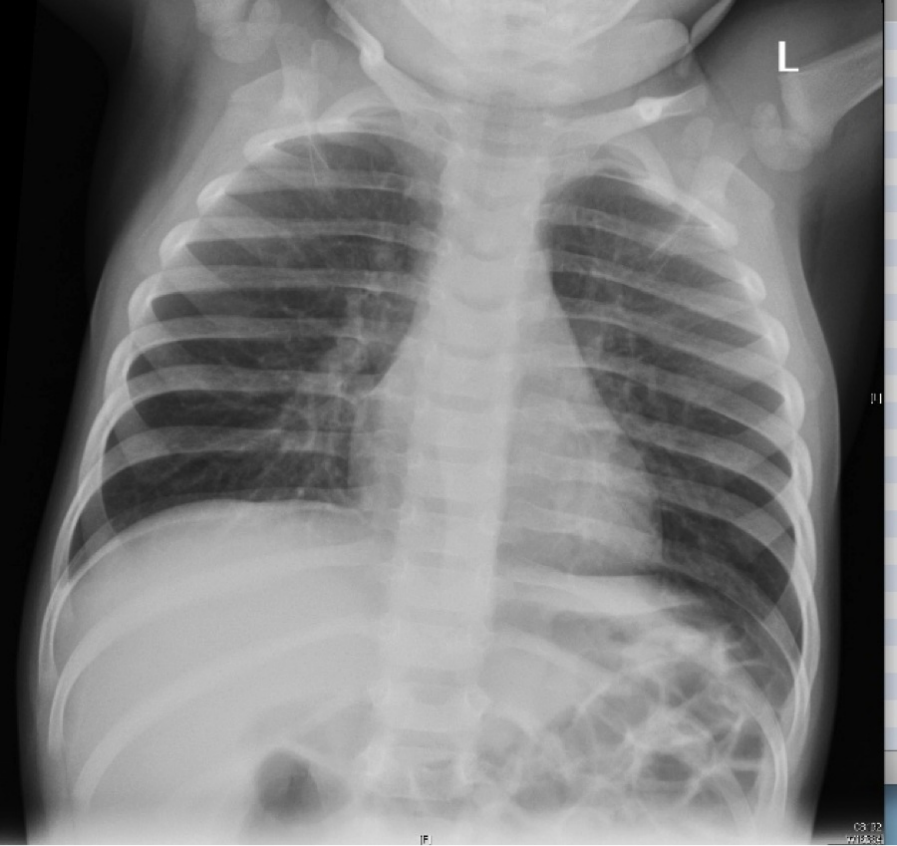
Chest X-Ray to be provided to trainee on request

Two trained emergency room physicians acted as instructors and aided in the scenario execution. In addition, technical staff operated the human patient simulator. One emergency room physician acted as the exercise lead ensuring technical staff followed the template (Table 1), provided supplemental learning materials as requested by the trainees (Figures 1-2, Table 2), and used practical experience from a clinical setting to troubleshoot any deviations or anomalies identified during the running of the case. The second emergency room physician used an a priori -developed assessment guide to note team performance, individual performance, and technical skills displayed in patient management. The report of the second instructor was used for formative assessment and debriefing the trainees following the simulation scenario (Table 3). Both emergency room physicians, following the completion of the scenario, debriefed the trainees.

**Table 1:** Technical outline required for mannequin programming and stepwise progression of infant trauma scenario.

Pre-Scenario		
You are an emergency room physician when a 1-year-old female infant is brought into the regional trauma center after a single vehicle accident in a minivan at highway speed. The emergency medical responders report to you that the child was restrained in an appropriate car sear. The paramedics also report that it took approximately 30 minutes to extract and transport the infant to the trauma center. The infant’s father was sitting next to her when the accident happened. He was declared dead at the scene and his wheelchair was found loose in the back of the minivan.		
Begin Scenario – Trainee enters the trauma room.		
Objective 1: Trauma Assessment		
Additional Scenario Details	Vital Signs/Physical Findings	Appropriate Trainee Action
Teacher as Paramedic: “One year old infant was found at the scene of a single vehicle accident. Mom was driving at 90km/h when she lost control on ice. The vehicle went into a ditch but did not roll”	Vital Signs: BP90/60 / HR130 / T35.5°C / RR30 / SpO2 98% RA	Order: Cardiac and SpO2 Monitor
	Physical Findings: Infant is limp and not crying	Order: 2 Large Bore IVs
	Physical Findings: Infant responds to pain but not voice	Order: Activate trauma team (see post-scenario didactics)
	Physical Findings: Pupils are sluggish	Trauma Assessment (Trainee Verbalizes): A – Airway is protected
	Physical Findings: Boggy scalp hematoma	Trauma Assessment (Trainee Verbalizes): B – Breathing not distressed
	Physical Findings: Closed fontanelle	Trauma Assessment (Trainee Verbalizes): C – Competent circulation
	Physical Findings: Brisk reflexes	Trauma Assessment (Trainee Verbalizes): D – Pupils sluggish
		Trauma Assessment (Trainee Verbalizes): E – Boggy scalp hematoma
		Use of Broselow Tape – Purple (10-11kg)
Teacher as Paramedic: “The infant was restrained appropriately. The father was found dead at the scene. His wheelchair was loose in the back of the van and it appears it hit the baby in the head. The mother is in the adult trauma center being assessed”	Vital Signs: BP90/60 / HR130 / T35.5°C / RR30 / SpO2 98% RA / Glu 6	Order: Labs (CBC, Electrolytes, BUN, Glucose, Creatinine, Liver Enzymes, Amylase/Lipase, INR, PTT, Blood Type & Screen)​
		Order: Warm blankets
		Order: EKG (See Figure 1)
		Order: FAST
		Order: Portable CXR (See Figure 2)
		Prepare for trauma resuscitation. Maintain C-Spine.
Objective 2: Trauma Resuscitation		
Additional Scenario Details	Vital Signs/ Physical Findings	Appropriate Trainee Action
Fluid Resuscitation	BP90/60 / HR115 / T36.5°C / RR30 / SpO2 98% RA	Initiate fluid resuscitation; normal saline 20ml/kg
Intubation	BP90/60 / HR115 / T36.5°C / RR30 / SpO2 98% RA	Rapid sequence intubation with appropriate agents (see post-scenario didactics)
If no fluid resuscitation and/or intubation	BP70/50 / HR90 / T36.5°C / RR30 / SpO2 80% RA	Initiate fluid resuscitation and intubation
If no warm blankets used	Temp remains 35.5°C	Order warm blankets
Objective 3: Reassessment and Management		
Additional Scenario Details	Vital Signs/Physical Findings	Appropriate Trainee Action
Reassess Vital Signs	BP90/60 / HR115 / T36.5°C / RR30 / SpO2 98% RA	Order CT head
Reassess Neurological Status	One pupil dilated	
Check Results of Ordered Tests	Labs: Normal (See Figure 3)	Order: Consider Further C-Spine Imaging (CT or MRI)
Verbal Radiology Report: “Left occipital subdural hematoma. Left intraparenchymal and associated mass effect”	Type and Screen: O-	Consult neurosurgery and PICU
	FAST: Negative	
	CXR: Normal	
	EKG: Normal sinus	
	CT Head: Intraparenchymal hemorrhage, subdural, mass effect (verbal report)	
Objective 4: Head Injury Management		
Additional Scenario Details	Vital Signs/Physical Findings	Appropriate Trainee Action
Intracranial hemorrhage described on verbal report from radiology	BP90/60 / HR115 / T36.5°C / RR30 / SpO2 98% RA	Initiate proper management of increased ICP: Mannitol 0.25-1 g/kg IV​
		Initiate proper management of increased ICP: Head of bed raised 30°
		Initiate proper management of increased ICP: Hyperventilation, target C02 30-35 mmHg
Head injury not addressed	BP70/50 / HR90 / T36.5°C / RR18 / SpO2 80% RA	
Baby consulted to neurosurgery and PICU.		
End Scenario		

**Table 2:** Laboratory results to be provided to trainee when requested from instructional staff.

CBC		
RBC	3.9 x 10^6^ μL	N
HgB	110 g/L	N
HCT	35%	N
MCV	80fL	N
MCHC	32%	N
Reticulocyte Count	--	N
INR	--	N
PTT	35 sec	N
Blood Type and Screen	O-	N
Electrolytes		
Na	135 mmol/L	N
Cl	105 mmol/L	N
K	3.7mmol/L	N
Mg	1.8 mmol/L	N
Ca	2.4 mmol/L	N
PO4	1.8 mmol/L	N
Chemistry		
BUN	2 mmol/L	N
Glucose	6.0 mmol/L	N
Cr	28.1 μmol/L	N

**Table 3:** Assessment guideline used by emergency room physician for formative or summative assessment of trainee and detailed feedback during debriefing.

Scenario Assessment Checklist	Completed	
	Yes	No
History		
Adequate History from Paramedics		
Physical Findings Realized		
Infant limp and not crying		
Infant responds to pain but not voice		
Boggy scalp hematoma		
Closed fontanelles		
Brisk Reflexes		
Proper Initial Actions		
Use of Broselow Tape (Purple 10-11kg)		
Objective 1: Trauma Assessment		
Order cardiac monitor		
Order SpO2 monitor		
Activate trauma team		
Order 2 Large Bore IVs		
Order Labs		
Order EKG		
Order Warm Blankets		
Order Portable CXR		
Order FAST		
Maintenance of C-Spine		
Trainee Verbalizes Trauma Assessment		
A – Airway is protected		
B – Breathing is not distressed		
C – Competent Circulation		
D – Pupils Sluggish		
E – Boggy Scalp Hematoma		
Objective 2: Trauma Resuscitation		
Order warm blankets		
Initiate fluid resuscitation (NS 20ml/kg)		
Rapid Sequence Intubation with appropriate agents		
Objective 3: Reassessment and Management		
Reassess vital signs		
Reassess neurological status – notice one pupil dilated		
Order CT Head		
Check results of ordered tests		
Realizes CT Head Radiology Report		
Consider further C-Spine Imaging (CT or MRI)		
Consult Neurosurgery and PICU		
Objective 4: Head Injury Management		
Order Mannitol 0.25-1g/kg IV		
Raise head of bed 30°		
Hyperventilate to CO2 30-35mmHg		
Conclusion		
Supportive care until Neurosurgery and PICU arrive		

### Pre-scenario briefing 

A pre-briefing session was held with all trainees preceding the start of the scenario. A team lead for the case was identified and the roles of each trainee were outlined. The roles of the technical staff and instructors were explained to the trainees. Scenario limitations relating directly to technical issues of the mannequin and availability of resources were reviewed with the trainees.

The nature of simulation exercises requires that a fiction contract be employed – an agreement between participants, instructors, and technical staff to proceed as if the simulation was real, while simultaneously acknowledging it was not. During the pre-scenario briefing the fiction contract was reviewed with all participating of the exercise. A mutual understanding of any contentious points was reached prior to the beginning.

Lastly, the trainees were informed that the nature of assessment for the scenario was strictly formative and that the results were to be used for self-directed learning and ongoing skills development. There exists the possibility of using simulation exercises as an evaluation tool for academic purposes using an objective-based checklist as outlined in Table 3. 

### Case

This simulation case involved a one-year-old infant patient presenting to a pediatric emergency department following a single motor vehicle collision. The patient was restrained in an appropriate car seat when the driver of the vehicle lost control on ice at 90 km/h. The infant’s father was sitting next to her in the vehicle and was declared dead at the scene. The father’s wheelchair was found loose in the back of the vehicle as well. Other information, if requested by the participants, could be provided by the instructor based on using their previous clinical experience.

The scenario was set in a pediatric hospital resuscitation bay with full access to a defibrillator, airway equipment, and a stocked resuscitation cart. The simulation case began with the patient connected to cardiac monitors displaying a full set of vital signs. Medications used in rapid sequence intubation were readily available. Depending on the nature of the group, one or more confederates can be recruited to play the part of a nurse, paramedic, or physician. The entire scenario was completed in a stepwise fashion as per the outlined in Table 1. The simulation technician ensured the mannequin responded appropriately to given or disregarded treatments.

During the scenario, an assessment guideline was used to assess trainees’ performance and identify any other points be addressed in the debriefing session. Both the lead instructor and the assessing instructor participated in the debriefing session.  

### Debriefing

Each trainee participated in a formalized debriefing session following the conclusion of the scenario. The debrief session was organized such that the trainee-to-instructor ratio was in favor of trainees (i.e., more trainees than instructors). An individualized approach to the debriefing session ensured that trainees could speak openly about any problems, technical difficulties, or any other issues that may have presented themselves during the course of the scenario. The session used a hybrid debriefing model that couples frame discovery [11-12] the 3D model of debriefing [13] was used.

The structure of the debriefing session started with a reaction phase that progressed into an inquiry and advocacy phase and ended with a didactic teaching and learning phase.

The Reaction Phase

This phase capitalized on the emotions of the students during the scenario and immediately following. A group discussion format was used to identify emotions experiences and normalize the reactions that various people experience.

The Inquiry and Advocacy Phase

This phase focused on how the objectives were handled during the course of the scenario and whether the trainees focused their attention in the right direction during the management of the patient. Specific areas that were addressed in this phase included:

1) Trauma assessment,

2) Age appropriate assessment of neurological status - AVPU responsiveness scale (alert, responsive to verbal stimulation, responsive to painful stimulation, and unresponsive),

3) Intubation and choice of RSI agents,

4) Maintenance of situational awareness during entire scenario without focus on one specific aspect. 

The trainees’ handling of specific objectives was addressed in a non-threatening manner that focused on frame discovery [12]. Trainees were solicited on what they felt they performed well on and which aspects they felt needed improvement. Following this, any inconsistency between the trainees' own identified learning needs, and the learning needs identified by the instructor were reconciled to a mutual agreement.

Didactic Teaching and Learning Phase

The final phase of the debriefing session utilized a didactic approach to address specific knowledge gaps that were identified, as well as to provide an overall standardized delivery of knowledge surrounding the handling of trauma in an infant (Table 4). 

**Table 4:** Post-scenario didactic objectives and suggested discussion points. This table is provided as an outline only to address the objectives of this particular case. The physicians conducting the post-scenario didactics should address the clinical details with the trainees, as well as any other questions or issues that may arise during the scenario.

Objective	Discussion Points
Trauma assessment using ATLS principles of ABCDE	The approach to the trauma patient should be organized and prioritized as per the ATLS principles. The use of the Broselow tape can assist the physician in determining the normal range of vital signs, as well as the correct dose of medications/fluids/electricity and the correct size of equipment commonly used in resuscitation.
AVPU neurological assessment in infants	Neurological assessment in children can be challenging, especially to the physician who is not primarily a pediatrician. The child’s developmental stage is an important consideration. The AVPU system of evaluation is a practical approach that can be easily applied to any developmental stage. A = alert, V = responds to verbal stimuli, P = responds to painful stimuli, U = unresponsive. It is also important to note that some pediatric emergency physicians use a modified Glasgow Coma Scale, as the motor component is predictive of adverse morbidity and mortality.
Projected course of severe head injury	In this particular case, the child has suffered a severe head injury and the projected course is to coma and possible death. The trainees should recognize that early airway management is vital, as is blood pressure control and initiation of cerebral edema management. Early consultation with a neurosurgeon is key. A discussion addressing other aspects of head injury management, such as mannitol, hyperventilation and raising the head of the bed is prudent. The possibility of concurrent injury, specifically c-spine injury, should be highlighted as well. As with any patient who has suffered multiple injuries, the management needs to be prioritized appropriately so that the most emergent injuries are dealt with first. These decisions should be made in consultation with the trauma team. For example, in this case, imaging of the c-spine should be pursued but can wait until the patient has stabilized and control of the ICP has been obtained. The type of imaging (ie. CT or MRI) may depend on local availability.
Intubation and drugs of rapid sequence intubation	A review of intubation technique and the drugs used is helpful to most trainees. A few key points relating to pediatrics patients should be highlighted. The use of atropine as a pre-medication to prevent/lessen hypotension is common in pediatrics; however, there is a lack of strong evidence to support this practice. Many physicians choose to use atropine in infants and young children, but not in older children. Choice of induction agent and paralytic agent should be addressed. The belief that ketamine can raise ICP has been disputed and trainees should be aware that this is a viable option in head injury. The anatomy of a child has some bearing on intubation. The epiglottis is relatively larger and floppy, making a straight blade laryngoscope (or similar video device) the preferred choice for most physicians. The larynx lies anterior and superior in comparison to adults and the narrowest point is the subglottic area hence the use of an uncuffed tube in young children.

## Discussion

Trauma situations can be technically difficult, emotionally charged, and medically complex, and if not managed appropriately, the projected course to mortality can be quite steep. Trauma situations can present in a multitude of ways, with no situation mimicking another. While the array of situations is variable, the control of trauma patients is premised on the principles outlined in the ATLS training course. With this in mind, the assessment and management of trauma for any situation becomes standardized among different trainees and institutions. The unique feature of pediatric trauma is the decreased level of prevalence in which trainees get to train on pediatric patients. This makes simulation training activities in the pediatric trauma setting extremely valuable.

The specific learning objectives of this simulation scenario focused on:

1) Trauma assessment using ATLS principles of ABCDE,

2) AVPU neurological assessment in infants,

3) Projected course of trauma situation,

4) Intubation and drugs of rapid sequence intubation.

The use of an analytical progression to develop the case as outlined in Table 1 allowed for the simulation to change in a manner than is dependent on decisions made by the trainee. An initial instructor run through ensures that the case is on par with the level of performance expected from an emergency medicine resident in a trauma situation in the emergency department. This initial instructor run through serves the added benefit of ensuring all technical and procedural difficulties of the case are identified and addressed. Finally, a formalized debriefing mode coupled to a structured didactic session allowed the instructors to identify any knowledge gaps experienced by the trainee and provide supplemental information to improve in the future.

## Conclusions

The recognition, treatment, and management of trauma situations is an integral part of an emergency medicine residency program. While trauma situations, especially those of infants, can be very demanding, proper training in a controlled setting can be used to curb the stress trainees experience in these situations and ultimately lead to be better patient outcomes. It is shown that the use of simulation to repeatedly practice a task results in improvement and proficiency on that task in the future [14]. We have presented a procedure designed to aid trainees in the completion of an infant trauma simulation-based scenario. In addition to the stepwise algorithm, an integrated session incorporating a practical simulation experience, didactic teaching, and a structured debriefing used to train emergency medicine trainees is outlined. 
